# An innovation for microstructural modification and mechanical improvement of TiAl alloy via electric current application

**DOI:** 10.1038/s41598-019-41881-z

**Published:** 2019-04-02

**Authors:** Zhanxing Chen, Hongsheng Ding, Ruirun Chen, Jingjie Guo, Hengzhi Fu

**Affiliations:** National Key Laboratory for Precision Hot Processing of Metals, School of Materials Science and Engineering, Harbin Institute of Technology, Harbin, 150001 China

## Abstract

In this article, microstructural evolution during the solidification of Ti-48Al-2Cr-2Nb with current density, as well as the formation mechanisms, are discussed, along with the impacts on microhardness and hot compression properties. The applied electric current promotes the solidification from the α primary phase to a largely β solidification in Ti-48Al-2Cr-2Nb. With an increase in supercooling, the solidification process have a tendency to change from an α-led primary phase to (α + β)-led primary phase. The primary dendrites, grain size, and lamellar spacing show a tendency to decrease first before increasing with increasing current density. Microhardness and high-temperature yield strength increase with a decrease in primary dendrite spacing, grain size, and lamellar spacing. Correlations between primary dendrite spacing, lamellar spacing, microhardness, yield strength, and current density are described by a fitting formula. An increase of α_2_ phase, due to the application of electric current, results in improved microhardness. The yield strength of Ti-48Al-2Cr-2Nb alloy increases linearly with microhardness. Yield stress increases with a decrease in microstructure parameters, in accordance with the Hall–Petch equation. The predominant modification mechanism with electric current application for TiAl solidification is the variation of supercooling and temperature gradients ahead of the mush zone due to Joule heating.

## Introduction

The solidification process of melts controls the crystal growth morphology and, ultimately, the columnar to equiaxed transition (CET). The application of an external energy field to metal liquid during solidification is a new way to change the behavior of nucleation and crystal growth and to modify the structure and enhance the mechanical properties of the solid. These fields, such as ultrasonic^1^, electromagnetic^2,3^, gravitational^4^, and electric current^5^, have shown to have great effects on the structural features and quality of cast metal. Processing technologies involving electric current are efficient, economical, and environmental methods of microstructural modification and have gained great attention in materials science, due to the resulting significant grain refinement, homogeneity and performance improvement. While advancements have been made in the studying of the solidification behavior of the melt pool with electric energy and the evolution of microstructure in various alloys, such as Sn alloy^5^, Al alloy^6^, cast iron^7^, and stainless steel^8^, there has been no report on experiment in which electricity is applied to assess the effect during the solidification process of high melting point and reactive alloy, such as TiAl.

TiAl alloys have attracted extensive interest among industrial companies for aeroengine applications due to the stringent requirements for higher thrust-to-weight ratios and greatly enhanced fuel efficiency^1,4,9^. TiAl are being used in the compressor part of aerospace gas turbine engines and the low pressure turbine part, because of its excellent mechanical properties. However, low processibility hinders the wide spread application of cast TiAl alloys^1,4,9^. Several strategies, such as the lowering of Al content to achieve fine lamellae structure, by adding rare earth elements such as Yttrium^10^ and Er^11^ to modify the microstructure, and the introduction of an external energy field to casting alloys^4^, were used to solve these problems. We show here that refining TiAl alloys with electric current application leads to a fine-grained microstructure, and gives rise to a higher ductility and improved deformability.

This paper reports the solid-liquid interfacial morphology, evolutional microstructure and mechanical performance of Ti-48Al-2Cr-2Nb alloy via direct electric current application. The mechanisms describing the influence upon electric current application on TiAl melt are discussed in detail. The correlation between microstructure and microhardness, and the correlation between microstructure and compressive deformation at high-temperatureare discussed.

## Results and Discussion

### Effect of electric current on solid-liquid interfacial morphology

**Figure 1:**
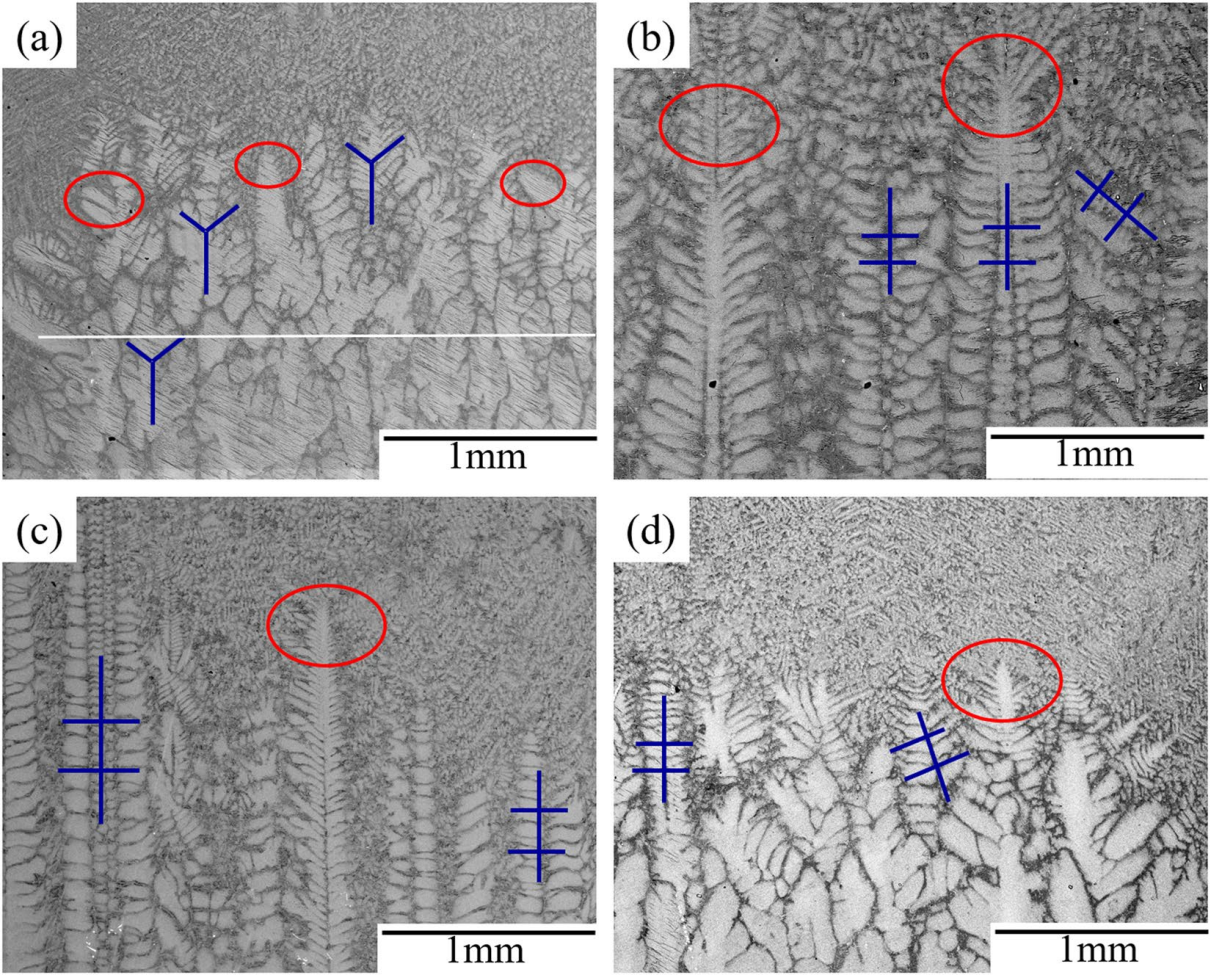
Solid-liquid interfacial morphology of Ti-48Al-2Cr-2Nb relating with current densities: (**a**) 0 A/m^2^, (**b**) 32 × 10^3^ A/m^2^, (**c**) 64 × 10^3^ A/m^2^, (**d**) 96 × 10^3^ A/m^2^.

Figure 1 presents solid-liquid interfacial morphology of Ti-48Al-2Cr-2Nb with different current densities under a steady withdraw speed (*V* = 10 μm/s). The reference specimen without current application (Fig. 1(a)) presents a dominance of coarse cellular-dendritic columnar crystal growing along the direction of the temperature gradient; the coarse cellular-dendritic growths are shown in the red ovals. The primary phase-α precipitates, as validated by the six-fold symmetry (color-coded with blue) in dendrite morphology of the α phase^12^.

The application of a lower direct current density ranging from 32 × 10^3^ A/m^2^ to 64 × 10^3^ A/m^2^ generates intensive dendrite morphology at the top and a reduced primary dendritic spacing, which increases with an increase in current intensity (Fig. 1(b,c)). The primary dendrite spacing decreases to 2 × 10^−4^ m (71.4% decrease), from 7 × 10^−4^ m in the reference sample, until the current density is 64 × 10^3^ A/m^2^, exceeding which the primary dendrite arm spacing starts to increase.

The tendency to solidify as a β-led primary phase can be observed with the application of, with the dendrite morphology changing from a six-fold symmetry to a four-fold symmetry. Moreover, the primary dendritic spines are perpendicular to the secondary dendrites in the cubic β phase^13^. The primary arms tend to split and form more ramifications. The dendritic structures are coarse after the density of electric current reaches 96 × 10^3^ A/m^2^ (Fig. 1(d)).

**Figure 2:**
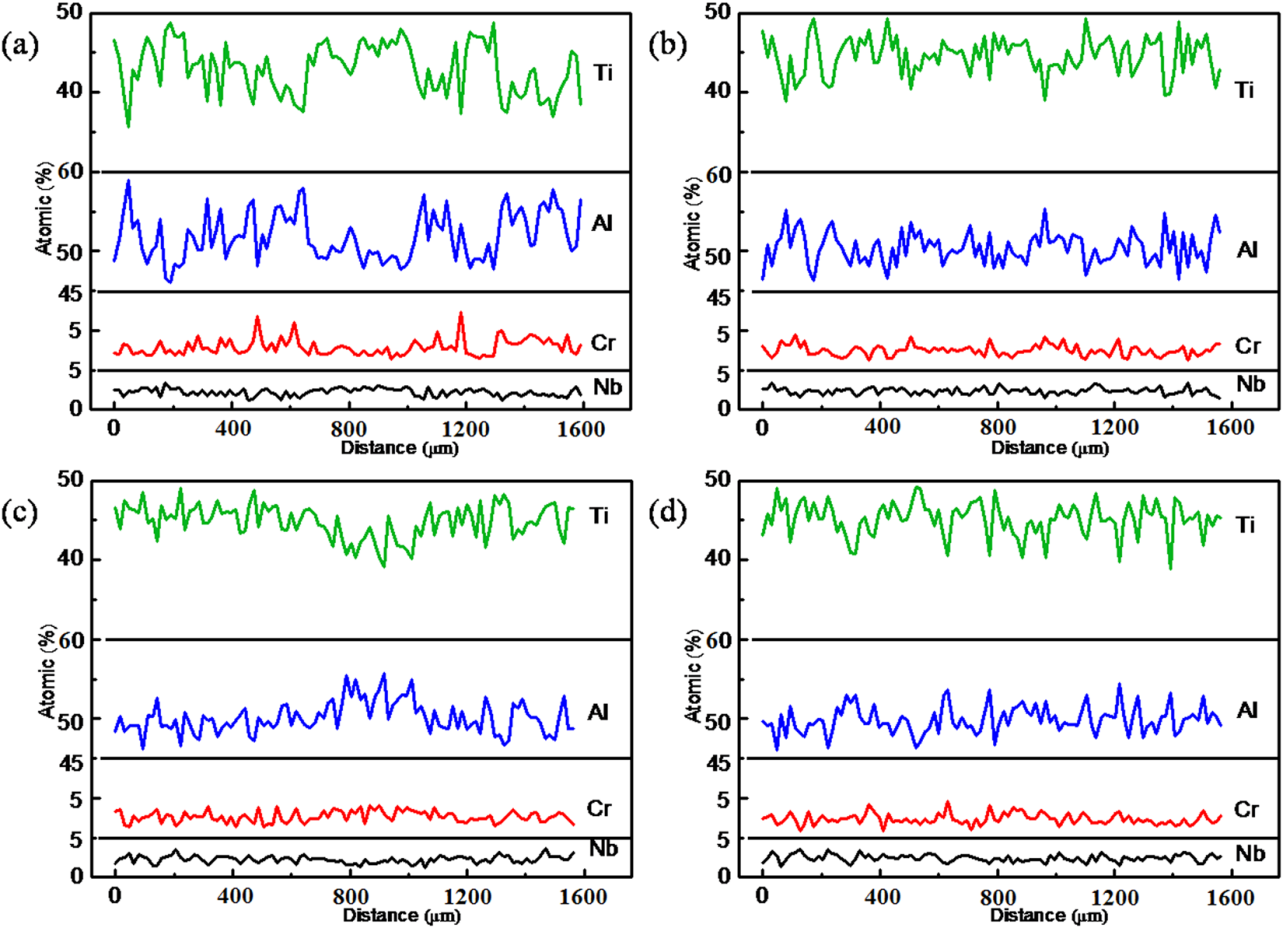
Distribution of elements in Ti-48Al-2Cr-2Nb solidified with electric current at the interface of liquid-solid in solid: (**a**) 0 A/m^2^, (**b**) 32 × 10^3^ A/m^2^, (**c**) 64 × 10^3^ A/m^2^, (**d**) 96 × 10^3^ A/m^2^.

Figure 2 shows concentrations of the constituent elements along the selected lines at 1 mm from the interface of liquid-solid in the solid of Ti-48Al-2Cr-2Nb directionally solidified with various electric current densities (the drawn lines in Fig. 1). 200 points were analyzed for each specimen, respectively. The results of electronic probe analysis show that the distribution of elements in the Ti-48Al-2Cr-2Nb alloy are non-homogeneous, the solute microsegregation of Ti-48Al-2Cr-2Nb either in dendrite arms or in dendritical regions is severe in the absence of applying electric current. The dendrite arms in Ti-48Al-2Cr-2Nb are enriched with Ti and Nb and depleted with Al and Cr, while the interdendritic regions are enriched with Al and Cr and depleted with Ti and Nb. Applying electric current can improve microsegregation and give a homogeneous distribution of the chemical composition of Ti-48Al-2Cr-2Nb alloy. Under a current density of 32 × 10^3^ A/m^2^, the dendritic segregation becomes alleviated. When the current density continues to increase to 64 × 10^3^ A/m^2^, the dendritic segregation eased further. However, when applies electric current rises from 64 × 10^3^ A/m^2^ to 96 × 10^3^ A/m^2^, the segregation increased significantly again.

Under equilibrium solidification conditions, according to the highest interfacial growth temperature criterion^14^, the β phase of a multiphase alloy with a higher interfacial growth temperature prefer to nucleate as the prevenient phase in the solidification process, where the α phase is suppressed. The resistance of the solid phase is lower than that of liquid phase. On one hand, the Joule heat generated by electric current in the liquid phase is greater than that in the solid phase, increasing the temperature gradient and supercooling of the solid-liquid interface frontier. The supercooling increases with increasing current density, and achieves a maximum value. The variation of primary dendritic arm spacings (PDAS) *λ* can be fitted with^15,16^:

1 $$\lambda \propto {{G}_{L}}^{-y}\cdot {v}^{-x}$$

where *G*_*L*_ is the temperature gradient at the solid-liquid interface; *v* is the crystal growth rate; *x* and *y* are indices in various mathematical models. Large supercooling enhances the solidification transformation from α-led primary phase to β-led primary phase and will led to the refinement of the PDAS. On the other hand, electric current is prone to propagate through dendritic peak in solid-liquid two-phase fluid, thus the Joule heating induced by electric current in dendritic peaks is larger than that in lateral branches, raising temperature in the dendritic tip. The branch peak will sink when melting occurs, and will result in spontaneous branch refinement in high Joule heating branches.

As solidification progresses and the applied current density continues to increase, Joule heating leads to solid phase remelting, while heat and mass transfer takes place with melt flow, the overall cooling rate, supercooling and the temperature gradient of solid-liquid interface decreases, and thus primary dendrites and grain size increase again.

### Effect of electric current on metallographic structure

**Figure 3:**
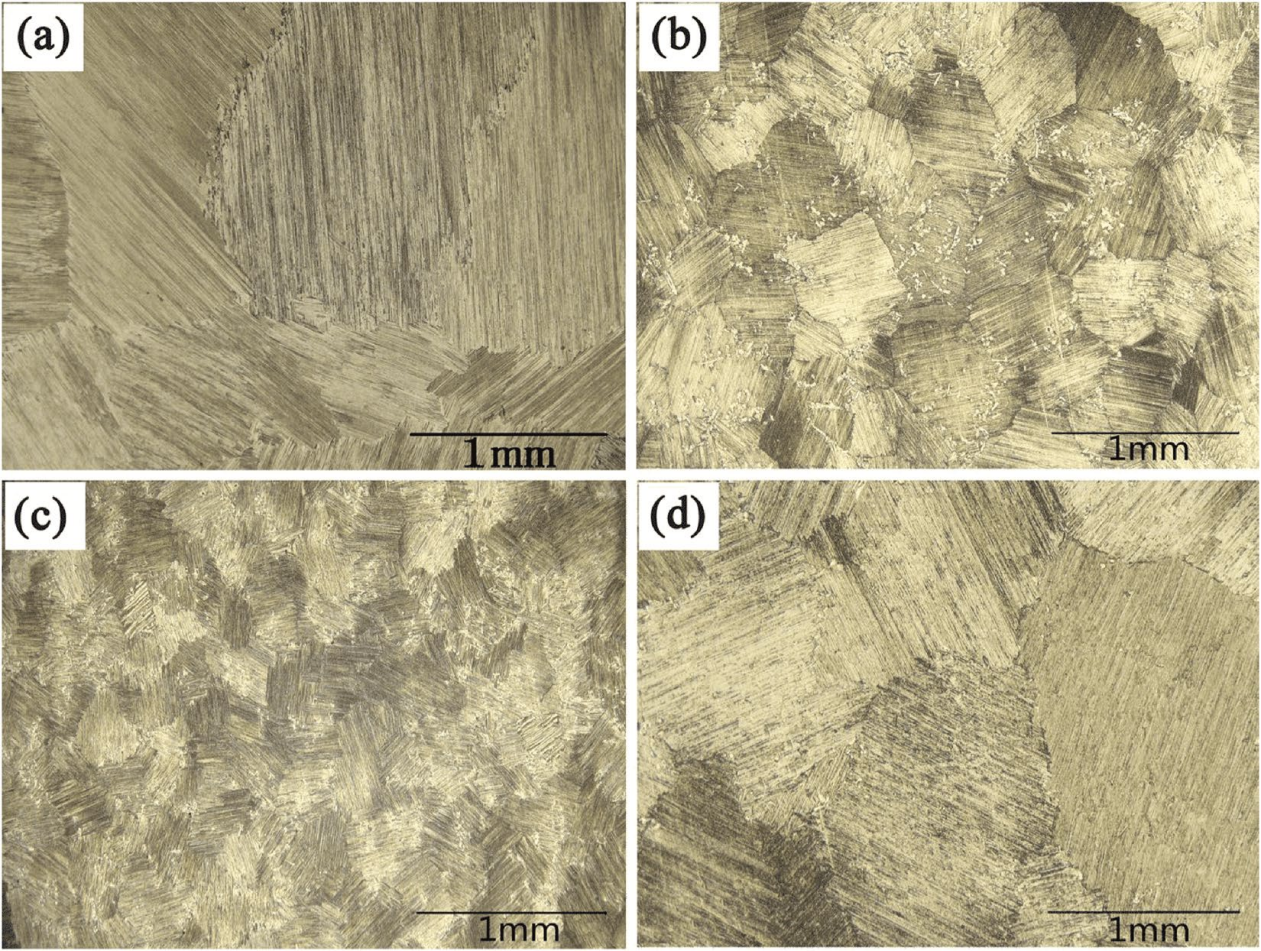
Metallographic structure of Ti-48Al-2Cr-2Nb solidified at different current densities of: (**a**) 0 A/m^2^, (**b**) 32 × 10^3^ A/m^2^, (**c**) 64 × 10^3^ A/m^2^, (**d**) 96 × 10^3^ A/m^2^.

The evolution of the metallographic structure in the transverse section at different direct electric current densities is shown in Fig. 3. Each grain in the microstructure presumably corresponds to a single grain of α, transformed from a larger β dendrite, which displays a similar full lamellar microstructure^17,18^. Therefore, the grain sizes and primary dendritic spacing also have the same variation trend. The sample without an applied electric current (Fig. 3(a)) shows a coarse and inhomogeneous grain size. The grain is refined and uniform when a lower current density ranging 32 × 10^3^~64 × 10^3^ A/m^2^ is applied (Fig. 3(b,c)). However, the grain becomes coarser significantly after the electric current density is increased to 96 × 10^3^ A/m^2^ (Fig. 3(d)). By measuring the grain sizes and fitting with linear regression analysis, the minimum grain size is found to be 115 μm at a current density of 64 × 10^3^ A/m^2^, about 23% of original size of reference sample.

Since the application of direct electric current is the only variable processing parameters performed during the solidification, it is believed that direct electric current is responsible for the grain refining effect observed in Fig. 3. Direct electric current results in alternative effects on the cylindrical specimens, leading to an increase in supercooling with low direct current and reduced supercooling with high direct current^19^. High supercooling and low temperature gradients are beneficial for grain refinement, as the probability of dendrite fragments that survive in the melt becomes larger with an increase in undercooled region^20^. As the current density goes from 0 to 64 × 10^3 ^A/m^2^, the dendrites are probably fused and ruptured owing to Joule heating, resulting in an increase in nucleation rate. The increased nucleation ratio arising from electric current contributes to grain refinement^21^. The Lorentz force acting on the solid liquid phase region has the potential to break dendrites into small debris owing to tear force^20,22^. Topical temperature and concentration profiles induced by flow within the solid-liquid region accelerate dendrite disruption by partial fusing of side arms, and the dendrite debris are transmitted afterwards by melt flow and subsequently nucleate in the inter-liquid region^23^. The forced melt convection due to electromagnetic stirring results in further grain refinement and also contributes to a reduced supercooling ahead of the mush zone^20^. The enlarged mush zone results in a decrease in the temperature gradient. Nuclei grow after exceeding a critical value according to thermodynamics mechanism; grains coarsen after current density reaches 96 × 10^3^ A/m^2^.

### Effect of electric current on lamellar space

**Figure 4:**
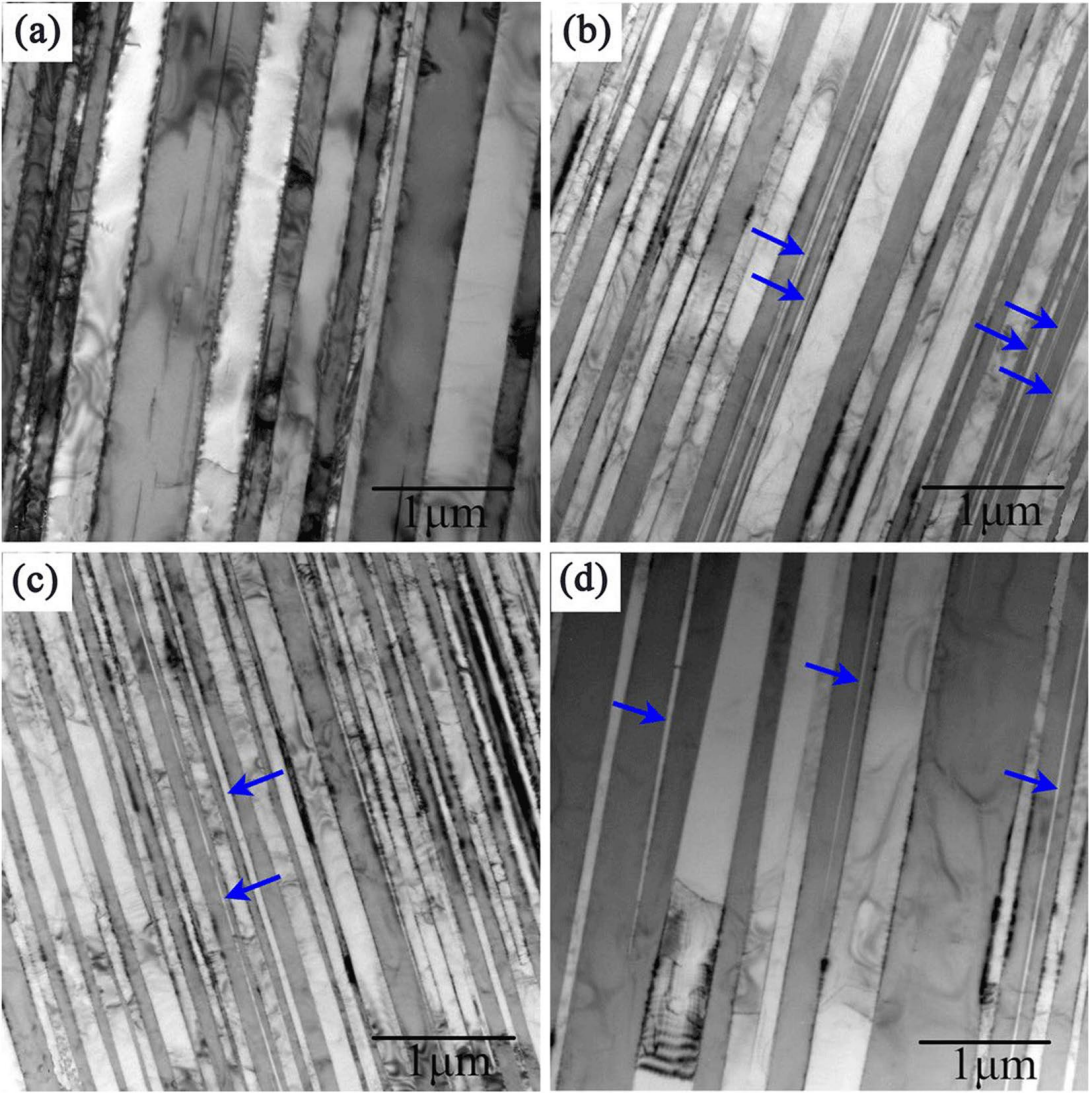
Lamellae structures of Ti-48Al-2Cr-2Nb solidified at different direct current densities of: (**a**) 0 A/m^2^, (**b**) 32 × 10^3 ^A/m^2^, (**c**) 64 × 10^3 ^A/m^2^, (**d**) 96 × 10^3 ^A/m^2^.

Lamellar structures of solidified TiAl alloys contain a single variant of α_2_ and two twin related variants of γ-phase. Figure 4 shows the microstructure of Ti-48Al-2Cr-2Nb directionally solidified at various current densities with other solidification parameters kept constant.

We observed an initial decrease followed by a subsequent increase in the lamellar spacing as current density increases, the same trend as the dendrites and grains. Tiny γ nuclei were precipitated from the Al-supersaturated α_2_-Ti_3_Al phase^24^, as indicated by the blue arrows (Fig. 4(b–d)). In addition, both α_2_/γ lamellae interfaces and γ/γ twin interfaces increase in quantity with the increase in direct electric current density. The precipitation of γ phase involves a hcp crystal (α_2_) transforming into an ordered fcc crystal (γ) accompanied by composition variation and dislocation gliding^25^.

γ lamellae is precipitated directly from the α phase matrix above the eutectoid temperature, thus the interlamellar spacing of the fully lamellar TiAl alloy is determined above the eutectoid transformation temperature. For the case of the same aluminum content, lamellar spacing is inversely proportional to the cooling rate. According to Tang^26^:

2 $${\rm{\lambda }}=\frac{{D}_{0}}{2kl}\cdot C(Al)\cdot \exp (-\,\frac{Q}{R{T}_{\alpha }})\cdot \frac{1}{{v}_{c}}$$

where *v*_*c*_ is the cooling rate, *D*_0_ is the interdiffusion constant of Ti and Al in the α phase, *k* is a constant, *l* is the length of the jog caused by the dislocation motion in α grains, *C*(*Al*) is a function associated with Al content of the alloy, *R* is the gas constant, *Q* is the activation energy, and *T* is the absolute temperature. Direct electric current has different effects on the supercooling, which can increase the supercooling with low direct current density, and reduce the supercooling with high direct current density.

TEM micrograph measurements indicate that α_2_/γ lamellar spacing *λ*_*m*_ strictly obeys log-normal distribution function *φ*(*λ*_*m*_):

3 $$\phi ({\lambda }_{m})=\frac{1}{{\sigma }_{2}\sqrt{2{\rm{\pi }}}}[-\frac{{(\mathrm{ln}{\lambda }_{m}-\mathrm{ln}\bar{\lambda })}^{2}}{2{{\rm{\sigma }}}_{2}^{2}}]$$

where $\bar{\lambda }$ is the averagelamellar spacing, *σ*_2_ is the variance under lognormal distribution^27^.

**Figure 5:**
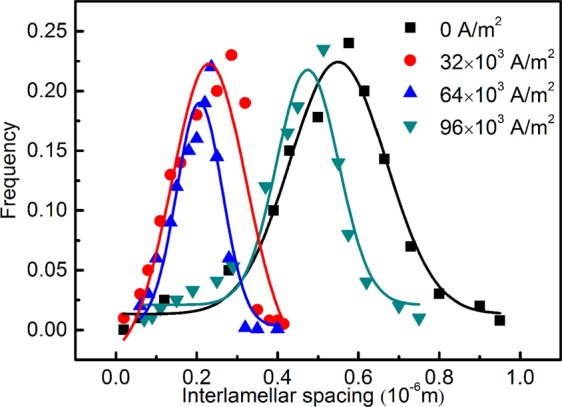
Log-normal frequency distribution of lamellar spacings for Ti-48Al-2Cr-2Nb ingots solidified with direct current.

Figure 5 summarises the log-normal frequency distribution according to the statistical assessment of lamellar spacings at different direct current densities. From the dispersion, the lamellae have been clearly refined and the uniformity of the scale is enhanced as the current density goes from 0 to 64 × 10^3^ A/m^2^.

**Figure 6:**
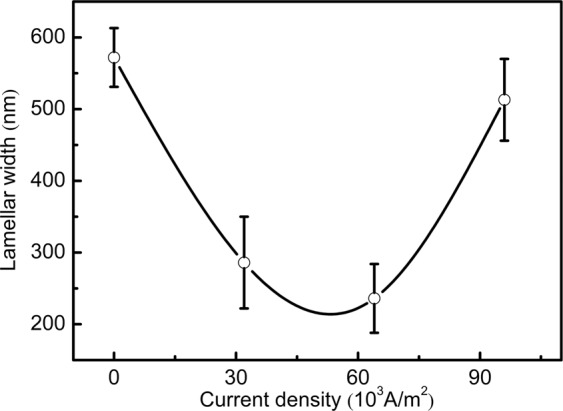
Dependency of average interlamellar spacing on direct current densities.

Figure 6 depicts the curve of the average lamellar spacings versus electriccurrent densities. The interlamellar spacing varies with the increase of current density in such a way that it decreases drastically up to 32 × 10^3 ^A/m^2^ and after that, increases moderately. The regression analysis of interlamellar spacing *λ*_*m*_ gives rise to the equation:

4 $${\lambda }_{m}=0.14{J}^{2}-14.2J+582$$

the fit coefficient of regression is r^2^ = 0.98, *J* is the direct current density, as previously stated.

### Effect of electric current on mechanical properties

**Figure 7:**
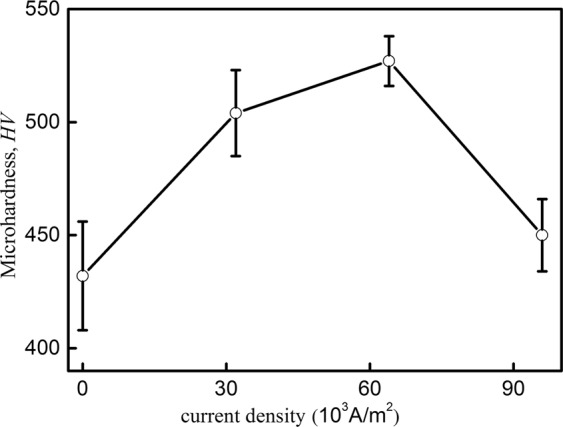
Vickers microhardness curve of Ti-48Al-2Cr- 2 Nb alloy following current densities.

Microhardness (*HV*) presents the resistance behavior for localized plastic deformation, densification and cracking of materials and is well correlated to tensile strength^28^. The property of microhardness is strongly dependent on the alloying component, processing conditions and structural parameters^29^. Figure 7 shows the Vickers microhardness curve of Ti-48Al-2Cr- 2 Nb alloy with different direct current densities. With increasing current densities, the value of microhardness first increases significantly, reaches the maximum of about 527 HV (roughly increased by 22% of the reference sample) at 64 × 10^3 ^A/m^2^, and then decreased. The change in hardness for the treated alloy can be understood by the reduction of the interlamellar spacing and grain refinement, explained by the Hall–Petch mechanism.

The γ phase presents lower microhardness value than α_2_ phase in TiAl alloys^29,30^. As current densities increase, the relative volume fraction of α_2_ phase increases and the formation of γ phase is reduced to various extents accordingly^31^.

**Figure 8:**
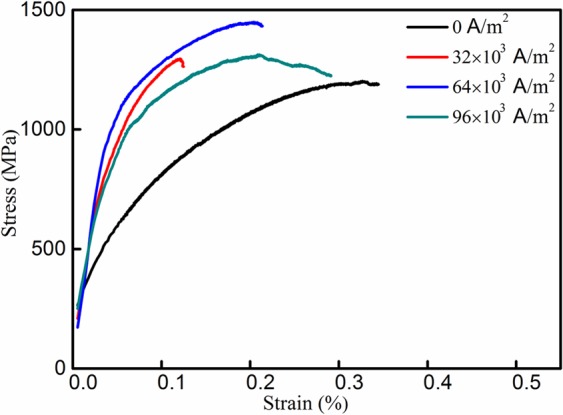
True stress-true strain relationships for Ti-48Al-2Cr-2Nb solidified at different current densities.

The true stress-true strain relationships used to measure the yield strength (σ_0.2%_) of solidified Ti-48Al-2Cr-2Nb under electric current are given in Fig. 8. It is apparent that the curves shift progressively toward the region of higher stress and lower strain rate with the increase in current density. The yield strength and fracture strength increase with the current density to a peak current density of 64 × 10^3^ A/m^2^; after which they fall with further increase in the current density. With the increase in current density from 0 to 64 × 10^3^ A/m^2^, yield strength increases from 432 MPa to 1196 MPa, and breaking strength increases from 1050 MPa to 1348 MPa. Yield stress increases with grain refinement and the decrease in interlamellar spacing. Conversely, ductility decreases with the increase in current density, which could be caused by contamination from the crucible^32–34^.

### Relationship between microhardness and yield strength

Based on the Hall-Petch relations, microhardness along with yield strength of conventional polycrystals are scaled by square roots of mean microstructural parameters of materials:

5 $${\sigma }_{y}={\sigma }_{0}+k{\lambda }^{-1/2}$$

where *σ*_*y*_ is the microhardness or yield stress, *σ*_0_ is material-dependent constant, *k* is Hall–Petch slope, and *λ* is microstructure length scale such as average grain size or interlamellar spacing^29,35,36^. According to Eq. (6), the microhardness (HV) and yield strength of materials change with the current densities (*J*) and microstructure length scales (*λ*_1_, *λ*_*m*_).

**Figure 9:**
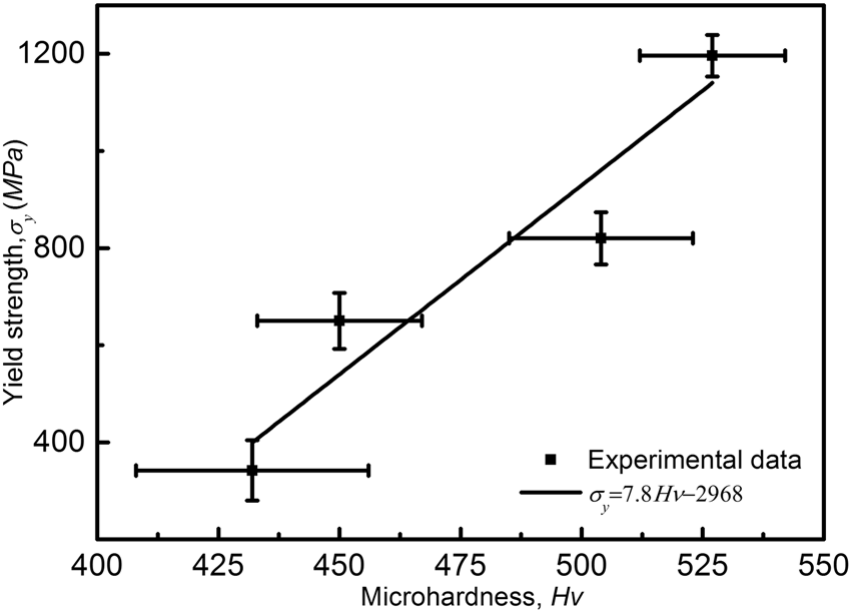
The Vickers microhardness dependence of the yield stress of Ti-48Al-2Cr-2Nb alloy solidified by direct electric current.

Hardness value depends more on the processing technique and attributes of the material, and hardness tests is an effective way for estimating other mechanical properties of metals^37^. Our experiments show that a linear functional relationship exists between values of yield strength and microhardness in Ti-48Al-2Cr-2Nb solidified with direct electric current. The fitted straight line for the dependence of microhardness on yield strength shown in Fig. 9 signifies the correlation between the increase in microhardness and performance enhancement. Empirical expression of the fitted line worked out by regressive analysis is given as

6 $${\sigma }_{y}=7.8Hv-2968$$

the regression coefficient of the fits r^2^ is 0.86.

The relationship between the yield strength and microstructure parameters manifests in the Hall–Petch equation with a bigger slope than previous research^29,32^, presenting enhanced fine-grain strengthening performance. It indicates that Vickers microhardness value can be used as a reference for controlling of the quality of TiAl alloys in a different processing technology. Eq. (6) shows that the yield strength of Ti-48Al-2Cr-2Nb alloy solidified with direct electric current, can be addressed possibly by the prediction of Vickers microhardness.

## Conclusions

In this work, a new electric current-aided technology solidified Ti-48Al-2Cr-2Nb alloy was investigated, giving following conclusions:

1. The applied electric current changes the solidification transition from α primary phase to a largely β solidification in Ti-48Al-2Cr -2Nb, motivating a transformation of an α-led primary phase to a β-led primary phase with an increase in current density. High current density would increase the β phase and push it to higher Al content region in TiAl phase diagram.
2. The evolution tendency of the primary dendrite spacing, grain size and lamellae spacing decrease first before increasing, and then increase gradually with an increase in current density.
3. By using direct electric current, the microhardness and yield strength of Ti-48Al-2Cr -2Nb are dramatically improved. Microhardness is linearly related to yield strength; yield strength and microstructure parameters obey the Hall–Petch equation, with a higher scale factor that signifies a higher strengthening efficiency of grain refinement.
4. The increase in nucleation due to the Joule heating is crucial in the mechanism of grain refinement for TiAl alloy at a lower current density, resulting in fused and ruptured dendrites. The grains coarsen at a higher current density due to a reduced supercooling and decreased temperature gradient ahead of the mush zone.

## Material and Methods

**Figure 10:**
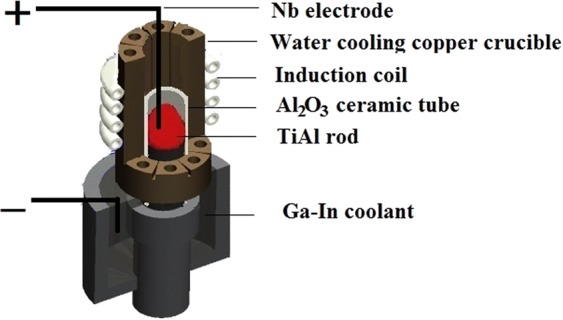
Schematic view of directionally solidified TiAl using direct electric current process.

The experiment raw material was Ti-48Al-2Cr-2Nb (at.%) alloy. A rod-like specimen cut from the mother ingot with a size of *Φ*14 × 90 mm was remelted in a Al_2_O_3_ ceramic mold by induction heating. The inside of the Al_2_O_3_ tube was coated with a Y_2_O_3_ skull to avoid contamination. The gap between the TiAl rod and Al_2_O_3_ tube gets filled with a homogeneous mixture of yttrium sol containing small Y_2_O_3_ particles. The solidification experiments were performed in a modified electromagnetic cold crucible directional solidification furnace in Fig. 10 as previously reported^31^. The heating temperature was measured by a W-Re thermocouple inserted into the melt in the course of the experiment. The TiAl rod was heated up to 1873 K with a temperature gradient of 15 K/mm and held at that temperature for 5 minutes. Two niobium wire electrodes were installed at both melted and unmeltedends of the TiAl bar respectively. The current in the bar was shown to be in the same direction as that of the bar withdrawal. The specimen was directionally solidified with a steady withdrawal velocity of 10 μm/s at a constant electric current density varying in the range 0 to 96 × 10^3^A/m^2^.

The solidified specimens were split axially along their mid-plane for metallographic investigations. The microstructure and initial crystal growth morphology were characterized by optical microscopy. The microsegregation of specimens before and after applying electric current was compared by means of electron probe micro-analyzer (EPMA). The lamellar structure was investigated by Transmission Electron Microscopy. The grain sizes (lamellar colony) from the transverse sections in the steady-state region (35 mm from the bottom part) of directional solidified specimens were measured by a quantitative metallography method. The primary dendritic spacings near the S/L interface (60 mm from the bottom part) of the vertical profile were measured by quantitative metallography method. Lamellar spacings of the steady-state region were measured from the TEM images by Image-Pro Plus. The measured values were averaged over multiple measurements.

Microhardness tests were conducted using a MICR0–586 type microhardness instrument under a loading of 1000 g for 20 s. High temperature compression performance was examined by Gleeble-1500D at a steady strain rates of 0.1 s^−1^, heating rate of 10 °C/s reaching 800 °C, the maximum true strain of 0.4. Before that, size of specimens *Φ*3 × 4.5 mm were prepared. To improve measurement precision, at least three repeatable trials were conducted.
